# CDK13-Mediated Cell Cycle Disorder Promotes Tumorigenesis of High HMGA2 Expression Gastric Cancer

**DOI:** 10.3389/fmolb.2021.707295

**Published:** 2021-08-26

**Authors:** Zhouying Wu, Min Wang, Feng Li, Feng Wang, Jianchao Jia, Zongqi Feng, Xue Huo, Jie Yang, Wen Jin, Rina Sa, Wenming Gao, Lan Yu

**Affiliations:** ^1^ Clinical Medical Research Center/Inner Mongolia Key Laboratory of Gene Regulation of the Metabolic Diseases, Inner Mongolia People’s Hospital, Hohhot, China; ^2^ Department of Pathology, Inner Mongolia People’s Hospital, Hohhot, China; ^3^ Departments of Cardiology, Hohhot First Hospital, Hohhot, China; ^4^ Department of Endocrine and Metabolic Diseases, Inner Mongolia People’s Hospital, Hohhot, China

**Keywords:** gastric cancer, cell cycle, CDK13, SR-4835, HMGA2

## Abstract

The inhibitor of CDK4/6 has been clinically used for treating certain types of cancer which are characterized by G0/G1 acceleration induced by the CDK4/6-RB1 pathway. On the contrary, the cell cycle–related molecules are abnormal in over 50% of the patients with gastric cancer (GC), but the efficiency of inhibiting CDK4/6 does not work well as it is expected. In our study, we found HMGA2 promotes GC through accelerating the S–G2/M phase transition, instead of G0/G1. We also found CDK13 is the direct target gene of HMGA2. Importantly, we analyzed 200 pairs of GC and the adjacent tissue and proved the positive relation between HMGA2 and CDK13; moreover, high expression of both genes predicts a poorer prognosis than the expression of single gene does. We explored the effect of the novel CDK12/13 inhibiting agent, SR-4835, on high *HMGA2* expression GC and found inhibition of both genes jointly could reach a satisfied result. Therefore, we suggest that inhibition of CDK13 and HMGA2 simultaneously could be an effective strategy for high *HMGA2* expression GC. To detect the expression of both genes simultaneously and individually could be of benefit to predict prognosis for GC.

## Introduction

Precisely regulated cell cycle maintains the normal cellular life, while uncontrolled cell cycle is one of the main features of all types of cancer. A series of complicated regulators, including cyclin-dependent kinases (CDKs), are involved in every detail of the cell phase transition of the cell cycle (Otto and Sicinski, 2017). Such CDKs as CDK4/6 are clearly researched (Harbour et al., 1999; Lazarov et al., 2002; Malumbres et al., 2004; Zhang et al., 2018a). Proved by the United States Food and Drug Administration, the CDK4/6 inhibitors have been commercially available and widely applied in some types of metastatic breast cancer, bringing the new landscape of treatment (Hortobagyi et al., 2016; Sledge et al., 2017; Slamon et al., 2018; Tripathy et al., 2018; Spring et al., 2020). Subsequently, the researchers have shed more light on not only the CDK4/6 inhibitor in many other types of cancer but also other members of CDKs (Min et al., 2018; Ding et al., 2020; Álvarez-Fernández and Malumbres, 2020).

CDK13, a transcription-associated CDK, was identified in 2001 and is known as the cholinesterase-related cell division controller as well as the regulator of the gene expression (Lapidot-Lifson et al., 1992; Marqués et al., 2000; Ko et al., 2001). CDK13 had been paid close attention in the children with certain types of congenital heart defects, and the heterozygous missense mutations of CDK13 would impair magnesium ion binding to ATP in these pediatric sufferers, yet no further studies were performed (Bostwick et al., 2017; Uehara et al., 2018; Hamilton and Suri, 2019; Novakova et al., 2019). The role of CDK13 in cancers, such as ovarian cancer and hepatocellular carcinoma, has been focused on since 2018; however, no underlining mechanism has been reported (Dong et al., 2018; Zeng et al., 2018; Wang et al., 2019). TCGA database showed that *CDK13* is amplified in different categories of cancer, indicating it could contribute to the tumorigenesis and development of cancer in humans. In 2019, it was reported that the triple-negative breast cancer might get benefit from the inhibitor of CDK12/CDK13, and the mechanisms were considered to be related to the enhancement of cell apoptosis by suppression of DNA damage response proteins and the cell cycle arrest induced by dysregulation of cell cycle checkpoint control proteins based on the RNA-seq data (Hopkins and Zou, 2019; Quereda et al., 2019; Tadesse et al., 2021). Although how the cell cycle checkpoint was regulated by these potential proteins has remained unclear, one point should be noticed: the inhibitor of CDK12/CDK13 might be a promising option for some types of cancer.

Gastric cancer (GC) has been in the lightening spot for years because of the high morbidity and low survival rate (Smyth et al., 2020). The uncontrolled cell proliferation of GC is mainly driven by the inordinate cell cycle progression. It was reported that the expression of the cell cycle–related molecules was abnormal in over 50% of the patients with GC (Min et al., 2018). Frustratingly, the inhibitor of CDK4/6, as a promising inhibitor to suppress the G0/G1 phase of the cell cycle, has not proved its efficiency as it is expected (Min et al., 2018). We suspect that there must be certain specific features as far as the GC cell cycle is concerned. Whether other types of CDK inhibitors could fight well against this specific cell cycle disorder of GC has not been explored, let alone the inhibitor of CDK12/CDK13.

High mobility group A2 (HMGA2) is a kind of non-histone chromosomal protein encoded by *HMGA2*. Its role is to modulate transcription by influencing the chromatin architecture through broadly binding to the chromatin and forming the multiprotein complex (Cleynen and Van de Ven, 2008). In the physiological condition, the expression of *HMGA2* is high during embryogenesis, but in most adult and differentiated tissues, the expression is almost undetectable (Zhou et al., 1996; Hammond and Sharpless, 2008; Nishino et al., 2008). Nevertheless, *HMGA2* is re-expressed in many types of cancer (Mansoori et al., 2021), and it could manipulate tumorigenesis, metastasis, and relapse *via* participating in cell cycle, apoptosis, angiogenesis, epithelial–mesenchymal transition, and chemoresistance (Zhao et al., 2018; Wu et al., 2019; Li et al., 2020a; Mansoori et al., 2020). HMGA2 could be considered a novel target gene for the precision therapy due to its vital role in cancer and the specific expression characteristics in different developmental stages (Zhu et al., 2017; Huang et al., 2018). As for its role in GC, HMGA2 is considered to be closely involved in the process of metastasis and the resistance to the medication (Wei et al., 2013; Hombach-Klonisch et al., 2014; Dong et al., 2017; Li et al., 2017; Sun et al., 2017). However, there has been rare attention to the relationship between the GC cell cycle and the expression of HMGA2.

In our study, we firstly found the GC tissues with high HMGA2 expression account for over 80% of GC. HMGA2 was then overexpressed based on the parental human GC cell lines, MKN-45 and MGC-803, and the proliferation of the cells accelerated. We found such acceleration was due to the shortened cell cycle phase transition. We further speculated that CDK13 might be the wirepuller when we analyzed our data from ChIP-seq and luciferase assay. This speculation was also reflected when we investigated the relationship between HMGA2 and CDK13 in 200 pairs of GC together with the adjacent tissue.

Furthermore, high expression of both genes predicts a poorer prognosis than the expression of single genes does. We explore the effect of the novel CDK12/13 inhibiting agent, SR-4835, on the high HMGA2 expression GC cells and consider the inhibition of both genes jointly could reach a satisfied result in high HMGA2 expression GC.

## Materials and Methods

### Cell Lines and Cell Culture

Human gastric cancer cell lines MKN-45 and MGC-803 were purchased from the National Infrastructure of Cell Line Resource. Cell line identities were confirmed by STR profiling. The cells were cultured in RPMI medium 1640 (Gibco, 11875-093) and DMEM (Gibco, 11965-092) supplemented with 10% fetal bovine serum (FBS) (Gibco, 10091-148) and 1% penicillin–streptomycin (Gibco, 15140-122) and were maintained at 37°C with 5% CO_2_.

### Establishment of the Stable Cell Lines

The sequence of sgRNA used in the study are listed in Table 1. Two single-guide (sg) RNAs targeting exon 1 (within the functional AT-hook domain) of *HMGA2* were designed. The pX330 vector (Addgene, 42230) was used to produce pX330-HMGA2-gRNA1 and pX330-HMGA2-gRNA2 plasmids. Lipofectamine LTX and Plus Reagent (Invitrogen, 2135022) were used in transfection. The single cells were sorted *via* flow cytometry (BECKMAN COULTER, MoFlo Astrios EQs, United States). The DNA and RNA were extracted, and PCR and Sanger sequencing were performed. The expression level of *HMGA2* was verified *via* RT-PCR and western blot assay.

**TABLE 1:** Sequences of primers, sgRNA, and siRNA used in the study.

Gene	Forward (5′ to 3′)	Reverse (5′ to 3′)
HMGA2-ORF	AGA​GAC​CCA​GGG​GAA​GAC​C	AGT​GGC​TTC​TGC​TTT​CTT​TTG​AG
HMGA2	ACG​TCC​GGT​GTT​GAT​GGT​G	TCT​TGC​TGC​TGC​TTC​CTG​G
CDK13	CAA​GCA​TAG​GAG​CCA​AGG​AGA​AG	AAT​CAG​CAA​GAA​GAC​ATC​GGA​GTT
TWIST1	GTC​ACA​ATG​CGG​AGC​CTA​AT	AAA​CCC​AGT​CCA​TGG​GAA​AG
HDAC6	GGGCGGTGATTGGTTGG	GAT​TCT​CTT​TCC​CTG​GTC​TTG​C
β-Actin	TCC​CTG​GAG​AAG​AGC​TAC​GAG​C	TGC​CAC​AGG​ACT​CCA​TGC​CCA​G

sgRNA	Forward (5′ to 3′)	Reverse (5′ to 3′)
HMGA2-1	CAC​CGG​TCC​TCT​CTT​CTG​AGG​CGC​T	AAA​CAG​CGC​CTC​AGA​AGA​GAG​GAC​C
HMGA2-2	CAC​CGT​GGG​GCG​GCA​GGT​TGT​CCC​T	AAA​CAG​GGA​CAA​CCT​GCC​GCC​CCA​C

siRNA	Sequence (5–3′)		
siRNA 1#	CGA​CGT​AGT​TTC​ATT​GGA​A		
siRNA 2#	GAG​AAA​TGG​TAG​CCT​TAA​A		
siRNA 3#	GCA​ATA​TCG​TCG​AAA​GTT​A		

*HMGA2*-overexpressed cells were established using pCMV6-Entry-*HMGA2* (OriGene Technologies, RC210804, China).

### Inhibition of CDK13

First, *CDK13* siRNA (si-h-*CDK13*, siB0804150912271, RIB BIO) or negative control siRNA (siR *NC*, siN000001-1-5, RIB BIO) was transfected into MKN-45 and MGC-803 using Lipofectamine® RNAiMAX Reagent (Invitrogen, 13778-150), respectively. Second, SR-4835 (TargetMol®, T8325/2387704-62-1) was used as the CDK13 inhibitor and added into the cell culture at 60 nM. The siRNA of CDK13 are listed in Table 1.

### RT-PCR and Western Blot

Total RNA from the cells was extracted using Trizol™ Reagent (Invitrogen, 15596018). Reverse transcription was performed using PrimeScript™ RT reagent Kit with gDNA Eraser (TaKaRa, RR047A). RT-PCR was performed using TB Green® Premix Ex Taq™ II (Tli RNaseH Plus, Takara, RR820A) on the CFX96™ Real-Time System (BIO-RAD). "The primer sequence used in the study are listed in Table 1.

The cells were lysed in RIPA (Solarbio, #R0020) containing a protease inhibitor cocktail (Millipore, 539136). The protein concentration was determined using the Pierce™ BCA Protein Assay Kit (Thermo Scientific, 23227). Specific antibody–protein complexes were detected with the ECL-PLUS Kit (Thermo, M3121/1859022), and the images were captured *via* a gel imaging system (GE Healthcare Life Scientific, Amersham Imager 600). The primary antibodies are as follows: anti-HMGA2 (CST, #5269S), anti-CDK13 (Invitrogen, VB2774502), and anti-GAPDH (Santa Cruz Biotechnology, sc-32233).

### Cell Proliferation *In Vitro* and *In Vivo*

The cell proliferation was recorded *via* an IncuCyte live-cell imaging system (Essen Bioscience, IncuCyte S3 2018B) for seven consecutive days. Meanwhile, cell viability was also determined *via* CellTiter 96 Non-Radioactive Cell Proliferation Assay Kit (Promega, G4001).

The animal experiments were approved by the animal ethics committee. Female NOD/SCID mice (7–8 weeks old, No. SCXK (Jing) 2016-0006) were purchased from Beijing Vitonlihua Experimental Animal Technology Co., Ltd. (Beijing, China), and were raised abiding by the principles of animal welfare. The processed cells were injected into the right subcutaneous axilla of the mice. The volume of the xenografts had been measured and calculated every week till the mice were sacrificed. The xenografts isolated from each group were measured and recorded.

### Cell Cycle and Apoptosis Assay

For detecting the cell cycle, the cells were treated with 2 mM thymidine (Sigma T1895), synchronized to the G1/S boundary, and stained using Click-iT™ EdU Alexa Flour™ 488 Flow Cytometry Assay Kit (Invitrogen, C10425). For detecting apoptosis, the cells were stained by FITC Annexin ⅤApoptosis Detection Kit Ⅰ (BD Biosciences, 556547) at the indicated days after seeding. The stained cells were detected by flow cytometry (BECKMAN COULTER, Navios, United States), and the data were analyzed using Kaluza Analysis Software (version 2.1).

### Chromatin Immunoprecipitation Following Sequencing Assay

According to the operating instructions, the ChIP procedure was conducted on *HMGA2*-overexpressed MKN-45 cells using HMGA2 Rabbit mAb (CST, #5269S). Histone H3 (D2B12) XP® Rabbit mAb (CST, #4620) acted as the positive control, and Normal Rabbit IgG (CST, #2729) acted as the negative control correspondingly. The results were verified by PCR. The primer we used was SimpleChIP® Human RPL30 Exon 3 Primers 1 (CST, #7014). IP efficiency was calculated with the percent input method. SimpleChIP® Plus Enzymatic Chromatin IP Kit (Magnetic Beads, CST, #9005) was used in the experiments.

The DNA was evaluated through a NanoPhotometer® spectrophotometer (IMPLEN, CA, United States) and Qubit® DNA Assay Kit in the Qubit® 2.0 Fluorometer (Life Technologies, CA, United States). The cDNA library was prepared and sequenced on Illumina’s NovaSeq platform to generate 150 base pair-end reads. The raw data were processed and mapped to the human reference genome (version hg19) using Bowtie (version 2.2.5). Peaks were identified by MACS (version 2.1.1.20160309) with *p* < 0.001. We calculated the read counts of genomic 500 bp regions for the treated sample and input sample. The UCSC RefSeq Genes database was used for peak annotation.

### Construction of Luciferase Vectors and Dual Luciferase Assay

GAPDH-PG04 and pEZX-PG04 vectors were purchased from GeneCopoeia. The recombined luciferase vectors were conducted and named pEZX-PG04-CDK13-P, pEZX-PG04-CDK13-D, and pEZX-PG04-CDK13-P/D, respectively. pCMV6-Entry-HMGA2 was used as the HMGA2 expression vector. The Gluc/SEAP dual-reporter vectors, together with *HMGA2* expression vectors, were transfected into *HMGA2*-knocked-out MKN-45 cells. The activities of Gluc and SEAP were measured using Secrete-Pair™ Luminescence Assay Kit (GeneCopoeia TM), and Gaussia luciferase activities were normalized to SEAP levels.

### Human Tissue Specimens and Immunohistochemistry

The procedures of human tissue sampling were approved by the Medical Ethics Committee of Inner Mongolia People’s Hospital. The enrolled patients signed the informed consent form beforehand. Surgical-resected tissues from the hospitalized GC patients were sectioned into slices of 5 μm thick, and 200 pairs of the tumor and the adjacent tissue were collected. All sections were stained with the primary antibodies of anti-HMGA2 (1:100, CST, 5269S) and anti-CDK13 (1:100, Invitrogen, VB2774502) using the IHC kit (for rabbit primary antibody, Bioss Antibodies, Cat: IHC001). The immunoreactivity of HMGA2 and CDK13 was evaluated based on the Quick Score System (Detre et al., 1995).

## Results

### The Proliferation and Tumorigenicity Were Different Among *HMGA2*-OE, *HMGA2*-P, and *HMGA2*-KO Cells

**FIGURE 1:**
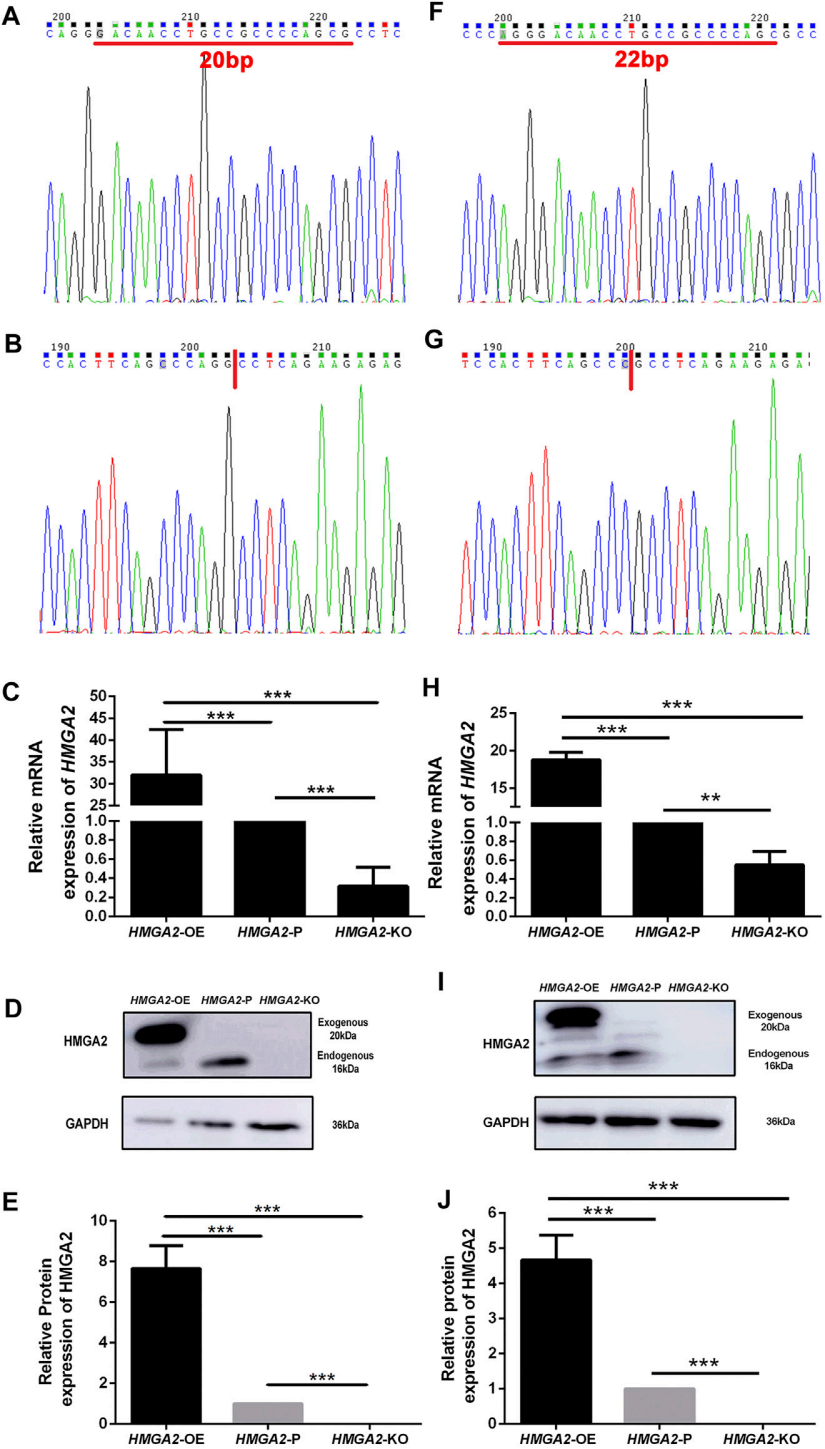
HMGA2 was knocked out in a biallelic manner and overexpressed successfully in two human gastric cancer cell lines. Sanger sequencing results of *HMGA2* in *HMGA2*-P MKN-45 **(A)**, *HMGA2*-KO MKN-45 **(B)**, *HMGA2*-P MGC-803 **(F)**, and *HMGA2*-KO MGC-803 **(G)**. Red lines in **(A)** and **(F)** indicate the deleted bases of *HMGA2* in *HMGA2*-KO MKN-45/MGC-803, respectively. The expressions of *HMGA2* in the mRNA level in *HMGA2*-edited MKN-45 cells **(C)** and MGC-803 cells **(H)** were detected. The expressions of HMGA2 in the protein level in *HMGA2*-edited MKN-45 cells **(D,E)** and MGC-803 cells **(I,J)** are shown. ***p < 0.001, Student’s t-test. The error bars represent SD.

*HMGA2* cells in parental MKN-45 and MGC-803 cells were knocked out in a biallelic manner *via* a CRISPER-Cas9-sgRNA gene editing system individually and named *HMGA2*-KO MKN-45 and *HMGA2*-KO MGC-803. Simultaneously, *HMGA2* cells were overexpressed based on the parental MKN-45 and MGC-803 cells. They were called *HMGA2*-OE MKN-45 and *HMGA2*-OE MGC-803, correspondingly. Sanger sequencing was conducted to investigate the gene knockout results, which showed 20-base pairs (bp) and 22-bp in *HMGA2*-KO MKN-45 and *HMGA2*-KO MGC-803 were deleted, respectively, implying the frame shift (Figures 1A,B,F,G). Subsequently, the expression of HMGA2 in *HMGA2*-KO MKN-45, *HMGA2*-KO MGC-803, *HMGA2*-OE MKN-45, and *HMGA2*-OE MGC-803 cells was detected using RT-PCR and western blot. The mRNA expression of *HMGA2* in *HMGA2*-OE MKN-45 cells was 30 times higher than the one in the parental cells. *HMGA2* in *HMGA2*-OE MGC-803 was 20 times higher than that in the parental MGC-803; on the contrary, the mRNA expression of *HMGA2* in *HMGA2*-KO MKN-45 cells was five times lower than that in the parental MKN-45. *HMGA2* in *HMGA2*-KO MGC-803 cells was two times lower than that in the parental MGC-803 (Figures 1C,H). As far as the protein expression of HMGA2 was concerned, the protein expression of HMGA2 in *HMGA2*-OE MKN-45 cells was seven times higher than that in the parental cells and the one in *HMGA2*-OE MGC-803 was five times higher than that in the parental MGC-803; on the contrary, the one was almost undetectable in both *HMGA2*-KO MKN-45 and *HMGA2*-KO MGC-803 cells (Figures 1D,E,I,J).

**FIGURE 2:**
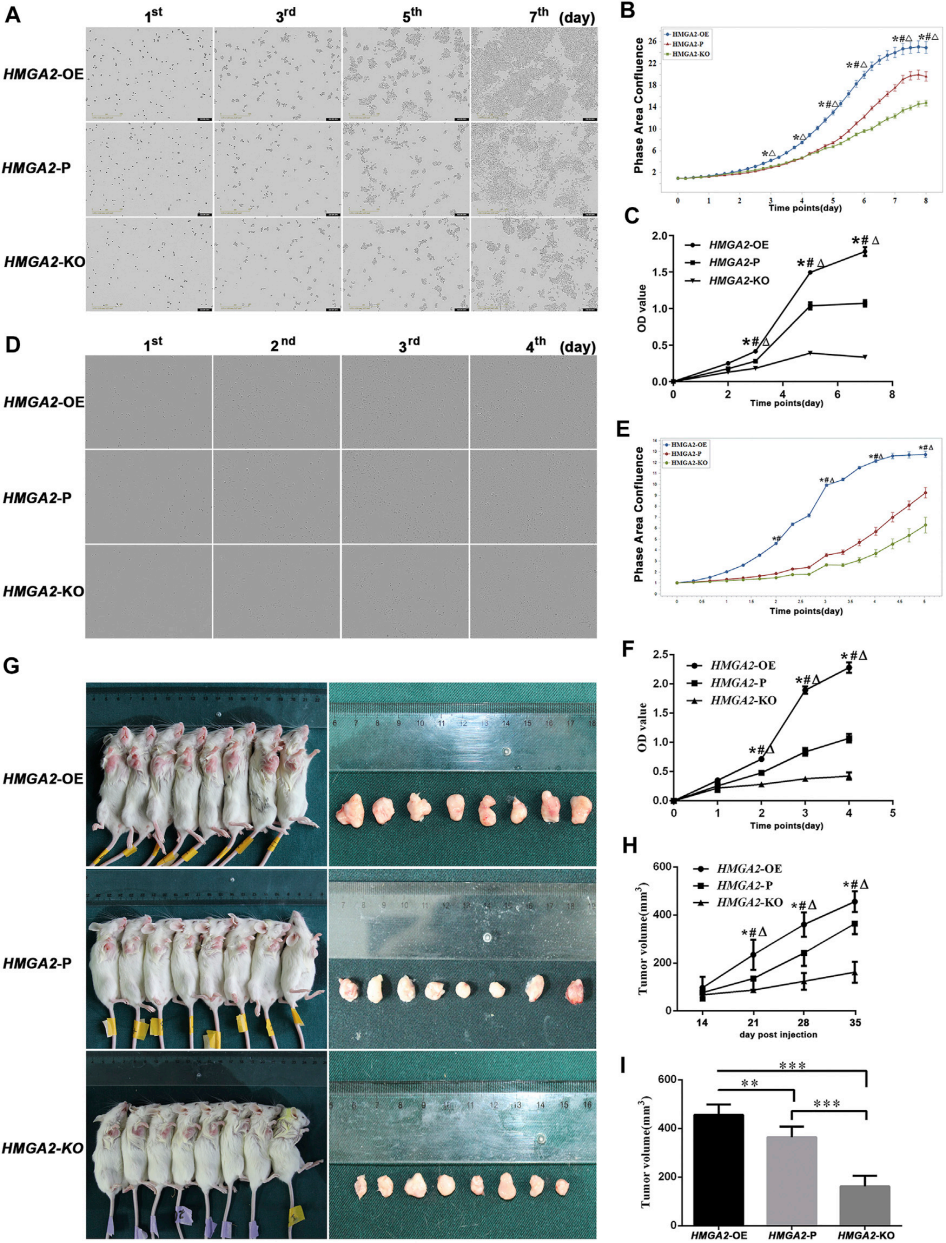
HMGA2 promotes the proliferation of the GC cells *in vitro* and *in vivo*. **(A)** Growth comparison of *HMGA2*-edited MKN-45 cells. The images in the vertical lines are the cell proliferation at different time (first, third, fifth, and seventh days). The pictures in the horizontal lines mean different groups: **upper**, *HMGA2*-OE cells; **middle**, *HMGA2*-P cells; **bottom**, *HMGA2*-KO cells. **(B)** Continuous proliferation records of *HMGA2*-edited MKN-45 cells *via* IncuCyte S3. Abscissa: consecutive days; ordinate: phase area confluence. The phase area confluences of the initial seeded cells in different groups were set as 1, respectively. Blue curve: *HMGA2*-OE cells; red one: *HMGA2*-P cells; green one: *HMGA2*-KO cells. **(C)** MTT assay to detect the growth of *HMGA2*-edited MKN-45 cells. *X*-axis: different days of cell viability; *Y*-axis: OD value. **(D)** Growth comparison of *HMGA2*-edited MGC-803 cells. Vertical lines: different time (first, second, third, and fourth days); horizontal lines: **upper**, *HMGA2*-OE cells; **middle**, *HMGA2*-P cells; **bottom**, *HMGA2*-KO cells. **(E)** Continuous proliferation records of *HMGA2*-edited MGC-803 cells *via* IncuCyte S3. Abscissa: consecutive days; ordinate: phase area confluence. The phase area confluences of the initial seeded cells in different groups were set as 1, respectively. Blue curve: *HMGA2*-OE cells; red one: *HMGA2*-P cells; green one: *HMGA2*-KO cells. **(F)** Growth of *HMGA2*-edited MGC-803 cells measured by the MTT assay. *X*-axis: different days of cell viability; *Y*-axis: OD value. **(G)** Tumor-bearing mice and the isolated xenografts. The NOD/SCID mice were inoculated subcutaneously under the right axilla with *HMGA2*-edited MKN-45 cells at the number of 2 × 10^6^ cells suspended in 100 µL PBS. The vertical lines are the sacrificed mice with xenografts and the isolated tumor correspondingly. **Upper line:** mice inoculated with *HMGA2*-OE MKN-45 cells; **middle line:** *HMGA2*-P MKN-45 cells; **bottom line:** *HMGA2*-KO MKN-45 cells. **(H)** Dynamic recording of the tumor volumes. The volumes of xenografts were measured each week on living mice. *X*-axis: different days; *Y*-axis: tumor volume (mm^3^). **(I)** Isolated tumor volumes of the NOD/SCID mice inoculated with different cells. *X*-axis: mice groups of *HMGA2*-OE, *HMGA2*-P, and *HMGA2*-KO cell inoculation, respectively; *Y*-axis: tumor volume (mm^3^). Symbols for **(B)**, **(C)**, **(E)**, **(F)**, and **(H)**: *, comparison of *HMGA2*-OE cells with *HMGA2*-P cells; △, comparison of *HMGA2*-OE cells with *HMGA2*-KO cells; #, comparison of *HMGA2*-P cells with *HMGA2*-KO cells; ^*,△,#^ *p* < 0.05. For **(I)**, **p < 0.01; ***p < 0.001, Student’s t-test. Error bars represent SD.

The proliferation ability of gene-edited cells and the parental cells was observed *via* IncuCyte S3 and MTT assay, respectively. As is shown in Figures 2A,B, no difference was found on day 0 because the number of planted cells was equal. On day 2, the descending order of proliferating speed was *HMGA2*-OE MKN-45, *HMGA2*-P MKN-45, and *HMGA2*-KO MKN-45, but without statistical significance. The growing speed of *HMGA2*-OE MKN-45 cells leapfrogged the parental cells and the *HMGA2*-KO MKN-45 cells from day 3 to day 8, and the parental cells transcended the *HMGA2*-KO MKN-45 cells from day 5 to day 8 with statistical significance, respectively (Figures 2A,B). The same trend was also proved *via* the MTT assay: such order began to show a statistical difference from day 3 to day 7 when the observation period was over (Figure 2C), suggesting that HMGA2 promotes the proliferation of the GC cells *in vitro*. Another GC cell line (MGC-803) also obtained the cell proliferating observation, presenting the same results as those in MKN-45 (Figures 2D–F).

To reassure the tumorigenic ability of the newly built cell lines, the *HMGA2*-edited MKN-45 cells were inoculated into the NOD/SCID mice. The xenografts were measurable at the 14th day in each group, and the tumorigenesis rate was 100%. The size of xenografts was too small to be compared among groups at the beginning until that of each group had the statistical difference at the 21st day. The descending order of xenograft size was *HMGA2*-OE, *HMGA2*-P, and *HMGA2*-KO MKN-45. When the time went by, such difference was increasingly statistically obvious (Figures 2G–I).

In a word, both the cellular phenotype and the xenograft animal experiment proved the success and stability of the newly gene-edited cell lines. The results confirmed that HMGA2 significantly increased cell proliferation capacity in a HMGA2-dependent manner both *in vivo* and *in vitro*.

### HMGA2 Shortened the S–G2/M Phase Transition and Influenced Little on Apoptosis

**FIGURE 3:**
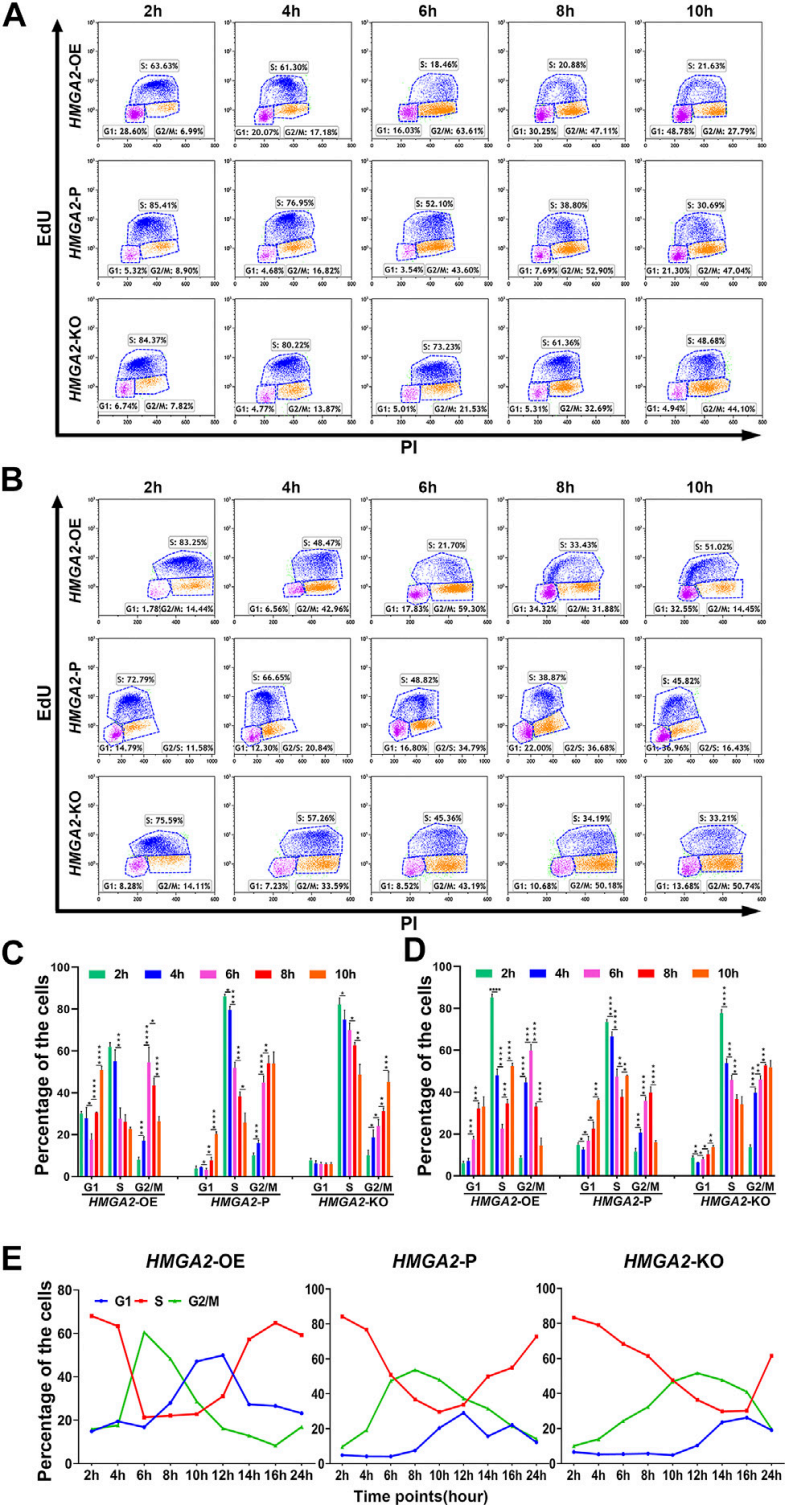
HMGA2 shortens S–G2/M phase transition of the cell cycle in two GC cell lines. **(A, B)** Cell cycle analysis at indicated time points *via* flow cytometry. Results from the **(A)** MKN-45 cells and **(B)** MGC-803 cells. Horizontal lines: **upper**, *HMGA2*-OE cells; **middle**, *HMGA2*-P cells; **bottom**, *HMGA2*-KO cells. Vertical lines: different time points after synchronizing the cells to the G1/S boundary. The time points are 2, 4, 6, 8, and 10 h. EdU negative and PI negative indicate the cells in the G0/G1 phase; EdU positive: cells in the S phase; PI positive: cells in the G2/M phase of the cell cycle. **(C,D)** Statistical histogram of the cellular percentage in each cell cycle phase at indicated time points. **(C)** MKN-45 cells and **(D)** MGC-803 cells. *X*-axis: different cell cycle phases of the different *HMGA2*-edited cells. *Y*-axis: percentage of the cells. **Left flock:** *HMGA2*-OE cells; **middle flock:** *HMGA2*-P cells; **right flock:** *HMGA2*-KO cells. *p < 0.05, **p < 0.01, ***p < 0.001, ****p < 0.0001, Student’s t-test. Error bars represent SD. **(E)** Dynamic percentage of the cells in each cell cycle phase recorded at continuous time. The cell cycle of MKN-45 cells was detected for a sequence of 24 h *via* flow cytometry after synchronized to the G1/S boundary, and the detection was conducted every 2 h. **Left:** *HMGA2*-OE MKN-45 cells; **middle:** *HMGA2*-P MKN-45 cells; **right:** *HMGA2*-KO MKN-45 cells. The red, blue, and green lines represent the proportion of cells in S, G1, and G2/M phases, respectively.

The cell cycle was compared among *HMGA2*-edited MKN-45/MGC-803 cells *via* flow cytometry, respectively. Taking MKN-45, for example, most of the *HMGA2*-OE cells spent 6 h to progress from the S phase to G2/M; when time increased to 8 and 10 h, more cells at the G2/M phase entered the G1 phase of the next cell cycle. In detail, as time went by, the number of *HMGA2*-OE cells at the S phase gradually dropped and reached the valley bottom at 6 h, while the ones at the G2/M phase increased and arrived at the peak at the same time point (6 h); on the contrary, the G1 cell number rose with the S and G2/M phase progression (Figure 3A upper, Figure 3C left side).

Interestingly, the number of S-phase *HMGA2*-P cells did not decrease to the bottom while the observing time period was about to complete. The decreasing trend, no platform, was observed as for the S-phase proportion while the G2/M-cell proportion was increasing adversely, but the dropping trend began to appear at 10 h, showing the G2/M cells began to progress into the next G1 phase (Figure 3A middle, Figure 3C middle). Such phenomenon was also observed in the *HMGA2*-KO groups; however, the proportion of the S-phase cells was 48.68% at 10 h, suggesting it still needs even longer time for most of the *HMGA2*-KO cells to progress into the next cycle (Figure 3A bottom, Figure 3C right side). The dynamic changes of the cell cycle observed among *HMGA2*-edited MGC-803 cells are consistent with what we observed in MKN-45 (Figures 3B,D).

To find out the exact time point when the number of S-phase cells in the *HMGA2*-OE, *HMGA2*-P, and *HMGA2*-KO groups was the least one, cell cycles were detected every 2 h for a sequence of 24 h (Figure 3E). Taking the percentage of S-phase cells in each group, for example, the ration of the cells in the S phase in the *HMGA2*-OE group began to decrease down to the valley bottom at the sixth hour, and after a flat for the next 4 h, the trend rose to the next peak, meaning the next cell cycle begins. However, such valley bottom was not the same as that in the other two groups, located at the 10th hour for *HMGA2*-P cells and the 14th hour for the *HMGA2*-KO cells, respectively. Thus, the portrait of how the expression level of HMGA2 influences the cell cycle was clearly presented. In brief, the lower the expression of HMGA2 in GC cells, the more the arrest of the cell cycle progression; furthermore, the arrest occurred in the S phase.

We investigated the apoptosis in *HMGA2*-edited MKN-45/MGC-803 cells, respectively. It turned out that there was no statistical significance among the groups (Supplementary Figure 1).

### CDK13 Was the Direct Target of HMGA2 in Gastric Cancer

**FIGURE 4:**
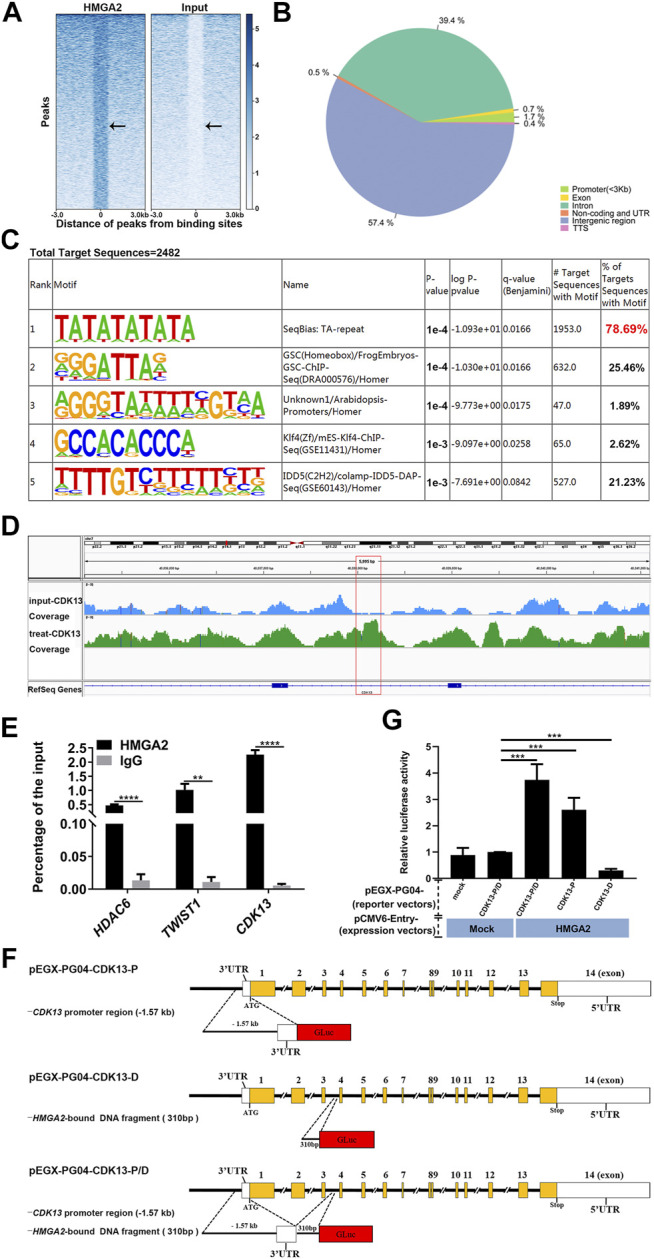
CDK13 is the direct target of HMGA2. **(A)** Heat map of ChIP-seq peaks. **Left part:** treated sample; **right part:** input control. The black arrow points to the HMGA2 binding sites. **(B)** Pie chart of the genomic distribution of HMGA2 binding sites. Blue part: intergenic region; dark green part: intron; light green part: promoter; yellow part: exon; remaining ones: TSS, non-coding and UTR. **(C)** Known motif enrichment results of the total target sequences immune-precipitated by HMGA2. **(D)** Enrichment analysis of the HMGA2 binding peaks at intron 3 of *CDK13*. The red box represents the specific binding site of HMGA2 in intron 3 of *CDK13*. The track signal is calculated by the average read count of a 500bp window. **(E)** Confirmation of the quantity of pull-downed *CDK13 via* ChIP-qPCR. *HDAC6* and *TWIST1* act as the positive controls. Abscissa: different genes; ordinate: percentage of the input. Black columns: DNA pulled down by HMGA2; gray one: DNA pulled down by IgG. **(F)** Schematic diagrams of recombinant Gluc/SEAP dual-reporter vectors. pEGX-PG04-CDK13-P **(the upper line):** the insert fragment cloned into pEZX-PG04 is the promoter region of *CDK13*, that is, the 1.57 kb upstream region from the first ATG codon on exon 1 of the *CDK13* gene; pEGX-PG04-CDK13-D **(the middle line):** DNA fragment immune-precipitated by HMGA2, that is, the 310bp fragment on intron 3 of the *CDK13* gene; pEGX-PG04-CDK13-P/D **(the bottom line):** promoter region of *CDK13* plus the DNA fragment immune-precipitated by HMGA2. **(G)** Relative luciferase activity of the reporter vectors with or without HMGA2. *HMGA2*-KO MKN-45, in which endogenous HMGA2 does not express, was transfected with the HMGA2 expression vector together with luciferase vectors in the following order: pEZX-PG04-Mock *vs* pCMV6-Entry-Mock; pEZX-PG04-CDK13-P/D *vs* pCMV6-Entry-Mock; pEZX-PG04-CDK13-P/D *vs* pCMV6-Entry-HMGA2; pEZX-PG04-CDK13-P *vs* pCMV6-Entry-HMGA2; and pEZX-PG04-CDK13-D *vs* pCMV6-Entry-HMGA2. Luciferase activities were normalized to SEAP levels, and the value of the pEZX-PG04-Mock *vs* pCMV6-Entry-Mock group was set as 1. **p < 0.01, ***p < 0.001, ****p < 0.0001, Student’s t-test. Error bars: SD.

To further elucidate the underlining mechanism by which HMGA2 induced the change of phenotype in GC cells, we performed ChIP-seq analysis of *HMGA2*-OE MKN-45 cells with anti-HMGA2 antibody. The results shown in Figures 4A–C confirmed the trustworthy findings of ChIP-seq. The Gene Ontology analysis of HMGA2-target genes showed that CDK13 was involved in the regulation of cell population proliferation (GO: 0042127). The enrichment of *CDK13* was also proved; as is shown in Figure 4D, compared with those in the input group, the peaks of the genes pulled down through HMGA2 are much higher in the district of *CDK13* intron 3, which was confirmed *via* ChIP-qPCR, and the results presented that the abundance of *HDAC6* and *TWIST1* which are the well-known target genes of HMGA2 and used as the positive control was much less than the abundance of *CDK13*; this can be explained as follows: HMGA2 bound efficaciously to a certain DNA area of *CDK13* (Figure 4E).

The Gluc/SEAP dual-reporter assays were performed to verify whether *CDK13* expression is directly regulated by HMGA2 in the live cells. Comparing the *HMGA2*-KO cells co-transfected with pEZX-PG04-CDK13-P/D and pCMV6-Entry-Mock to the cells co-transfected with pEZX-PG04-CDK13-P/D and pCMV6-Entry-HMGA2, the stronger luciferase activity in the latter one proved that HMGA2 directly upregulated the expression of CDK13 (*p* < 0.001). Comparing the cells co-transfected with pEZX-PG04-CDK13-P and pCMV6-Entry-HMGA2 to the cells co-transfected with pEZX-PG04-CDK13-P/D and pCMV6-Entry-HMGA2, the luciferase activity was much stronger in the latter one, suggesting the DNA fragment found by ChIP is probably the enhancer of *CDK13* (Figures 4F,G).

### HMGA2 and CDK13 Were Highly Expressed in Gastric Cancer and Related With Poorer Prognosis

**FIGURE 5:**
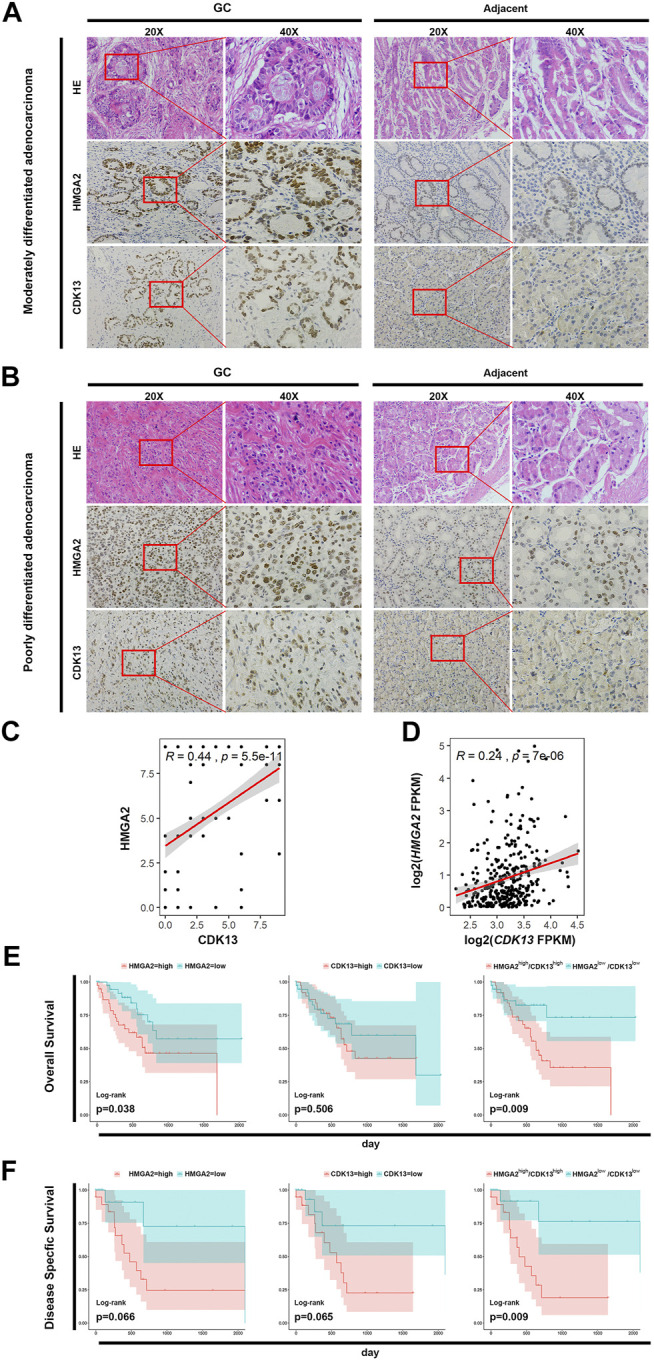
HMGA2 and CDK13 are associated with tumorigenesis of GC. **(A)** Expression of HMGA2 and CDK13 in the tissues of the patients with moderately differentiated STAD. The **left** two columns are the HE staining and immunohistochemical results of the GC tissue. The **right** two columns are those from the adjacent tissue. For both GC and the adjacent group, respectively: the first column, ×20; the second one, images magnified from the red frames of the **left** side, ×40. Compared with the corresponding location of the adjacent tissue, the morphological disorder of cell arrangement is obvious in the GC tissue although the glands exists, which is consistent with the characteristics of this type of GC. The brown colored nuclei in the images of the second and the third horizontal line suggest HMGA2 positive and CDK13 positive, respectively. Therefore, the expressions of HMGA2 and CDK13 in GC are much higher than those in the adjacent tissue. **(B)** Expression of HMGA2 and CDK13 in the tissues of the patients with poorly differentiated STAD. The arrangement of images is the same as that of A. The HE staining and immunohistochemical assay were also performed on the poorly differentiated GC. The cells are more disorderly growing, losing the normal glandular morphology, and the sizes of the cells are more varied compared with those in the moderately differentiated GC, suggesting the high grade of malignancy. Positive HMGA2 or CDK13 immunohistochemical staining (in brown) localizes mainly in the nucleus of the cells. Strong positive staining in GC tissues and weak positive staining in corresponding adjacent tissues are shown. **(C)** Pearson’s correlation between the expression of HMGA2 and CDK13 from the immunohistochemistry results of the 200 GC tissues was calculated. *X*-axis: expression of CDK13; *Y*-axis: expression of HMGA2; *R* = 0.44, *p* = 5.5e-11. **(D)** Pearson’s correlation between *HMGA2* and *CDK13* based on the RNA-seq results of the 416 STAD patients in TCGA database was calculated. *R* = 0.24, *P* = 7e-06. **(E)** Kaplan–Meier survival analysis for overall survival (OS). The expression of *HMGA2* and *CDK13* of 76 patients with tubular STAD was stratified to OS, respectively and simultaneously. **(F)** Kaplan–Meier survival analysis for disease-specific survival (DSS). The expression of *HMGA2* and *CDK13* of 44 patients with stage IV STAD was stratified to DSS, respectively and simultaneously. For **(E)** and **(F)**: **left**, expression of *HMGA2*; **middle**, expression of *CDK13*; **right**, expression of *HMGA2* and *CDK13* simultaneously. Pink color: high expression; blue: low expression.

The expression of both *HMGA2* and *CDK13* in the stomach adenocarcinoma (STAD) was much higher than those in the normal mucosae based on TCGA database (Supplementary Figure 2). To confirm such trends, cancer tissues as well as the adjacent tissues from 200 STAD patients were processed and the expression level of HMGA2 and CDK13 was explored *via* the immunohistochemical method, and the results are consistent with those from TCGA database (Figures 5A,B). The percentage of HMGA2 and CDK13 positive was approximately 80% in GC *vs* 25% in the adjacent tissue and 60% in GC *vs* 15% in the adjacent tissue, respectively (Table 2). The correlation of HMGA2 and CDK13 was statistically analyzed, and *R* = 0.44, *p* = 5.5e-11041 (Figure 5C). What we found in the GC patients was consistent with the results from analyzing TCGA database (Figure 5D).

**TABLE 2:** Expression difference of HMGA2 and CDK13 between GC and the adjacent tissue.

Percentage of positive expression	GC tissue (N = 200)	The adjacent tissue (N = 200)	*p*-Value
HMGA2	80% (160)	25% (50)	<0.001
CDK13	60% (120)	15% (30)	<0.001

How do *HMGA2* and *CDK13* affect the overall survival (OS)? Survival curves relating to the expression level of *HMGA2* and *CDK13* were analyzed based on TCGA database, respectively and jointly. In the patients with tubular STAD, the OS of the patients with either low *HMGA2* expression or low *CDK13* expression has more optimistic prognosis than the cases with high expression. The OS of the patients with both high *HMGA2* expression and high *CDK13* expression simultaneously was much poorer than those with low expression. Furthermore, the statistical significance of the OS difference between the simultaneous high expression of both genes and low expression was more obvious than the OS difference between the patients with high and low expressions of the single gene (Figure 5E). Such characteristic can also be seen in the disease-specific survival (DSS) analysis of the patients with stage IV of diffuse type STAD (Figure 5F). It could be inferred that the patients with high *HMGA2* and *CDK13* expression GC have poorer prognosis. Targeting HMGA2 and CDK13 associatively would be the possible promising new therapy to enhance the five-year survival rate of GC.

### Synergic Inhibition of HMGA2 and CDK13 Had the Most Suppressing Efficacy on the Growth of the Gastric Cancer Cells

**FIGURE 6:**
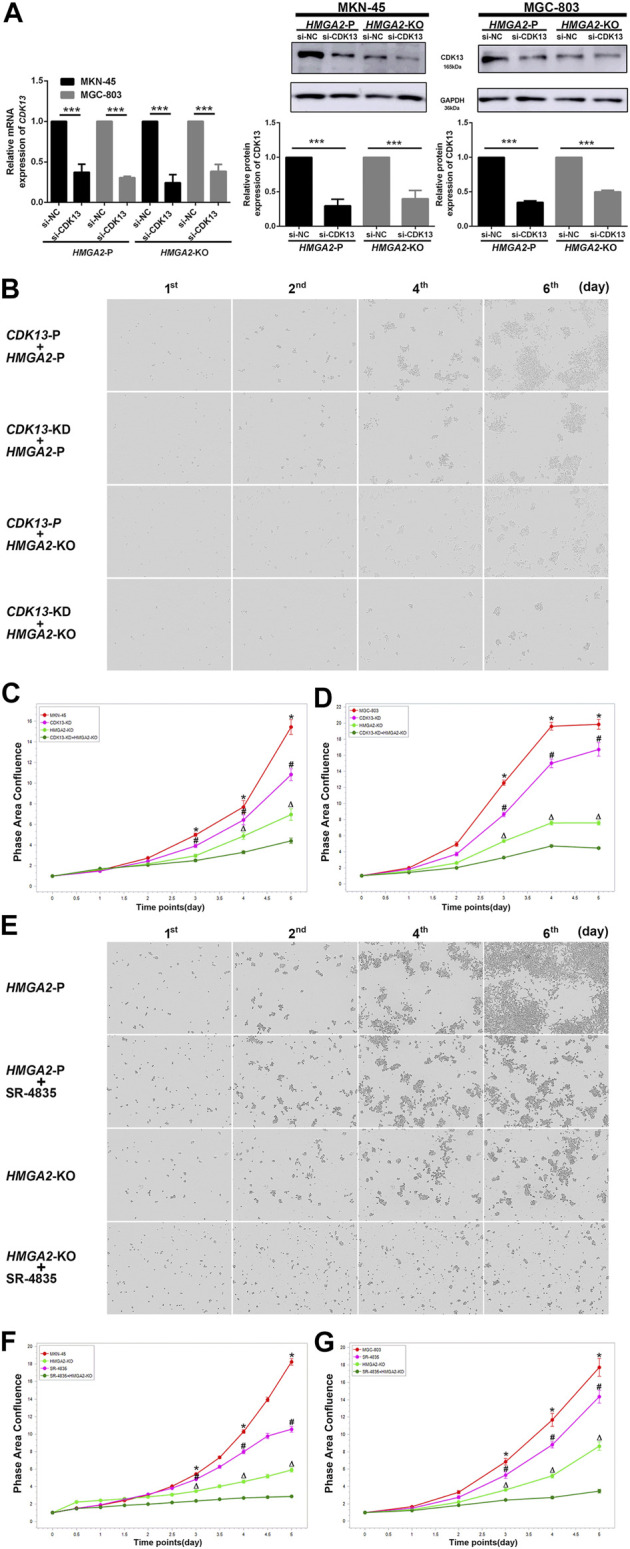
Inhibiting CDK13 and HMGA2 simultaneously suppresses the GC cells most. **(A)** Efficiency of knocking down CDK13 in two GC cell lines. **Left:** the efficiency was verified by RT-PCR. *CDK13* was knocked down based on *HMGA2*-P and *HMGA2*-KO MKN-45/MGC-803, respectively. *X*-axis: different cell groups. *Y*-axis: expression of *CDK13*. **Middle** and **right:** western blot results of the expression of CDK13 in *HMGA2*-P and *HMGA2*-KO MKN-45 and MGC-803, respectively. ***p < 0.001. **(B)** Proliferation of the MKN-45 cells in different groups. The images in the vertical lines are the cell proliferation at different time (first, second, fourth, and sixth days). The pictures in the horizontal lines mean different groups: ***upper***, *CDK13*-P plus *HMGA2*-P cells; second, *CDK13*-KD plus *HMGA2*-P cells; third, *CDK13*-P plus *HMGA2*-KO cells; ***bottom***, *CDK13*-KD plus *HMGA2*-KO cells. KD: knockdown; KO: knockout; P: parental. **(C)** Continuous records of the proliferation of the different gene-edited MKN-45 cells. **(D)** Continuous records of the proliferation of the different gene-edited MGC-803. For **(C)** and **(D)**: abscissa, consecutive days; ordinate, phase area confluence shown *via* IncuCyte S3. Red curve: *CDK13*-P plus *HMGA2*-P cells; pink one: *CDK13*-KD plus *HMGA2*-P cells; light green one: *CDK13*-P plus *HMGA2*-KO cells; dark green one: *CDK13*-KD plus *HMGA2*-KO cells. The phase area confluences of the initial seeded cells in different groups were set as 1, respectively. *: comparison of *CDK13*-P plus *HMGA2*-P cells with *CDK13*-KD plus *HMGA2*-KO cells; #: comparison of *CDK13*-KD plus *HMGA2*-P cells with *CDK13*-KD plus *HMGA2*-KO cells; △: comparison of *CDK13*-P plus *HMGA2*-KO cells with *CDK13*-KD plus *HMGA2*-KO cells; ^*,△,#^ *p* < 0.05. **(E)** Proliferation of the different treated MKN-45 cells. The images in the vertical lines are the cell proliferation at different time (first, second, fourth, and sixth days). The pictures in the horizontal lines mean different groups: **upper**, *HMGA2*-P cells; the second line, *HMGA2*-P plus SR-4835 treated cells; the third line, *HMGA2*-KO cells; **bottom**, *HMGA2*-KO plus SR-4835 treated cells. **(F)** Continuous proliferation records of the different types of MKN-45 cells. **(G)** Continuous proliferation records of the different types of MGC-803. Abscissa: consecutive days; ordinate: phase area confluence shown *via* IncuCyte S3. Red curve: *HMGA2*-P cells; pink one: *HMGA2*-P plus SR-4835 treated cells; light green one: *HMGA2*-KO cells; dark green one: *HMGA2*-KO plus SR-4835 treated cells. The phase area confluences of the initial seeded cells in the different groups were set as 1, respectively. *: comparison of *HMGA2*-P cells with *HMGA2*-KO plus SR-4835 cells; #: comparison of *HMGA2*-P plus SR-4835 cells with *HMGA2*-KO plus SR-4835 cells; △: comparison of *HMGA2*-KO cells with *HMGA2*-KO plus SR-4835 cells; ^*,△,#^ *p* < 0.05.

The *HMGA2*-KO MKN-45/MGC-803 cells together with their parental version were used to verify the anti-cancer efficacy of inhibiting HMGA2 and CDK13 associatively. They are as follows: *CDK13* knocked down only (*CDK13*-KD + *HMGA2*-P), *HMGA2* knocked out only (*CDK13*-P + *HMGA2*-KO), *CDK13*-KD together with *HMGA2*-KO (*CDK13*-KD + *HMGA2*-KO), parental (*CDK13*-P + *HMGA2*-P) MKN-45 and MGC-803. The efficiency of knocking down *CDK13 via* siRNA was confirmed by RT-PCR and western blot, and the efficiency was about 60% (Figure 6A). As expected, the growth of *CDK13*-KD + *HMGA2*-KO cells was suppressed the most; the ascending order of cell proliferation was *CDK13*-P + *HMGA2*-KO, *CDK13*-KD + *HMGA2*-P, and *CDK13*-P + *HMGA2*-P MKN-45/MGC-803 (Figures 6B–D). Subsequently, SR-4835 was used to inhibit CDK13 as an alternative method except siRNA. It turned out that no matter how CDK13 was inhibited, whether by SR-4835 or siRNA, the speed of the cell growth in different groups has maintained the same order (Figures 6E–G).

## Discussion

GC has been in the lightening spot for years because of the high morbidity and low survival rate (Smyth et al., 2020). HMGA2 has been reported as a GC-promoting gene, but how HMGA2 regulates the cell cycle in GC cells has not been illustrated clearly. Some researchers have proved that HMGA2 could promote the G1/S and G2/M phase transitions, respectively, in the ovarian cancer and leukemia (Malek et al., 2008; Zhang et al., 2018b). In our study, the overexpression of HMGA2 accelerated the S–G2/M transition in the GC cells, instead of the G0/G1 phase, which was consistent with the findings in leukemia (Tan et al., 2016). On the contrary, some inhibitors of the cell cycle are coming into clinical practice. For instance, CDK4/6 inhibitors are providing survival benefit to the certain types of breast cancer characterized by G0/G1 acceleration induced by the CDK4/6-RB1 pathway (Goel and Tolaney, 2019; Zhang et al., 2019). We explored the anti-proliferation effect of a CDK4/6 inhibitor, palbociclib, on *HMGA2*-edited GC cells and found the cells were insensitive to palbociclib (the data are not shown). This phenomenon was also confirmed by Ahrum Min et al. (2018) who reported that out of 10 human GC cell lines, only four were sensitive to CDK4/6 inhibitors and the other six were less sensitive or even insensitive (Min et al., 2018). Thus, we deduced that CDK4/6 inhibitors could not work well in some types of GC. We then carried out transcriptome sequencing, ChIP-seq, and luciferase assay to reveal the specific cell cycle–related regulator of S–G2/M phase transition in high *HMGA2* expression GC and found HMGA2 bound to intron 3 of the *CDK13* gene directly. This is the first study that elucidated the relationship between HMGA2 and CDK13. Accordingly, we suspect CDK13 might involve in the S–G2/M transition. Quereda et al. (2019) reported CDK12/CDK13 can significantly upregulate the S–G2/M–progressing genes (Quereda et al., 2019). Interestingly, the high expression of both *HMGA2* and *CDK13* jointly predicts a poorer prognosis in our research; therefore, choosing the right cell cycle inhibitors and jointly inhibiting CDK13 and HMGA2 might be an effective strategy for the high *HMGA2* expression GC.

CDK13 has been found to have the increasingly important role in cancer biomarkers and therapeutic targets in recent years (Dong et al., 2018; Zeng et al., 2018; Quereda et al., 2019; Wang et al., 2019), while it has detailed function and the underlying mechanism has not been clearly investigated. The amino acid identity between CDK13 and CDK12 is approximately 50%; furthermore, 92% of their main functional structures are the same (Greifenberg et al., 2016). Because CDK12 and CDK13 have the identical conserved kinase domains and activating partner (cyclin K), CDK13 has been studied as the companionship of CDK12 from the beginning, and the most light has been shed on CDK12, veiling the real face of CDK13 (Greifenberg et al., 2016; Fan et al., 2020; Tadesse et al., 2021). Thus, the function and the mechanism of CDK12 have been comparatively clear; moreover, the inhibitor of CDK12 was invented and has already entered the clinical trials (Blazek et al., 2011; Dubbury et al., 2018; Liang et al., 2020). However, there is no specific inhibitor for CDK13 until THZ531, a selective CDK12/13 inhibitor, was found in 2016 (Zhang et al., 2016). Unfortunately, THZ531 was not suitable for clinical use due to the problem of bioavailability and toxic off-target (Hopkins and Zou, 2019). The recently discovered SR-4835 (a selective dual inhibitor of CDK12/CDK13) exhibits excellent anti-cancer therapeutic effects, especially when combined with PARP inhibitors (DNA-damaging chemotherapy) or anti-PD-1 (checkpoint inhibition) (Quereda et al., 2019; Li et al., 2020b). Moreover, it is reported that the IC50 of SR-4835 for CDK12 and CDK13 is different (98 nM for CDK12 *vs* 4.9 nM for CDK13). Perhaps, the IC50 difference between them could be utilized for the independent study of CDK13. In our study, we also investigated the anti-cancer effect of SR-4835 at low concentration on high *HMGA2* expression GC, and the results showed the excellent anti-cancer effect, especially when combined with HMGA2 knockout. Therefore, our findings might provide the proof for selecting the type of cell cycle inhibitor in treating GC and the basic data for molecular classification of GC.

Accumulating evidence has reported the functional difference between CDK12 and CDK13 (Quereda et al., 2019; Tadesse et al., 2021). *CDK12* was not pulled down in the ChIP experiment in our study, enlightening the thought that CDK13 indeed shares some molecular function with CDK12, but CDK13 might have its own specific role and its unique mechanism, and CDK13 could be studied unbinding to CDK12. More research studies are needed to map the independent role of CDK13, which is the main task in our next study.

## Data Availability

The datasets presented in this study can be found in online repositories. The names of the repository/repositories and accession number(s) can be found below: GSE174442.
