# Supplementary material for: CDK13-Mediated Cell Cycle Disorder Promotes Tumorigenesis of High HMGA2 Expression Gastric Cancer
Source: Front Mol Biosci. 2021 Aug 26;8:707295. doi: 10.3389/fmolb.2021.707295 (PMC8427521; doi:10.3389/fmolb.2021.707295)
Supplement: Supplementary file 1 [file DataSheet1.doc]

**Supplementary Figure 1. HMGA2 does not induce the apoptosis in two GC cell lines.**

A: The apoptosis at the indicated time points in MKN-45 cells via flow cytometry. The horizontal lines: the upper line: *HMGA2*-OE cells; the middle line: *HMGA2*-P cells; the bottom line: *HMGA2*-KO cells. The vertical line: the different time points after the cell seeding: 3^rd^, 7^th^, 10^th^, and 14^th^ day; Annexin V (-) and PI (-): the live cells; Annexin V (+) and PI (-): the early apoptotic cells; Annexin V (+) and PI (+): the late apoptotic cells; Annexin V (-) and PI (+): the dead cells. B: The apoptosis at the indicated time points in MGC-803 cells via flow cytometry. C: Statistical histogram of the cellular percentage at various stages of apoptosis at the indicated time points in MKN-45 cells. The different color means the different groups corresponding to the images in A. There are no statistical differences among groups. D: Statistical histogram of the cellular percentage at various stages of apoptosis at the indicated time points in MGC-803 cells. ns: no statistical significance, two-way ANOVA. Error bars: SD.

**Supplementary Figure 2. The expression of *HMGA2* and *CDK13* was investigated from the TCGA database.**

The RNA-seq results of *HMGA2* and *CDK13* based on 415 STAD cases and 34 cases of the normal mucosae from TCGA database. The up left: Comparison of *HMGA2* in the normal tissue to that of the STAD cases; the up right: the expression of *HMGA2* in STAD cases with different age. Compare the expression of in the normal tissue to that in GC tissue, we found the expression of *HMGA2* in GC is much higher than that in the normal tissue, no matter which age group are concerned. It was worth noticing that the expression of HMGA2 and CDK13 in younger patients was higher than those in the patients from other age groups. The bottom left: Comparison of *CDK13* in normal tissue to that of the STAD cases; the bottom right: the expression of *CDK13* in normal cases and in STAD cases categorized by patients’ age.
