# Supplementary figures and images for: CDK13-Mediated Cell Cycle Disorder Promotes Tumorigenesis of High HMGA2 Expression Gastric Cancer
Source: Front Mol Biosci. 2021 Aug 26;8:707295. doi: 10.3389/fmolb.2021.707295 (PMC8427521; doi:10.3389/fmolb.2021.707295)

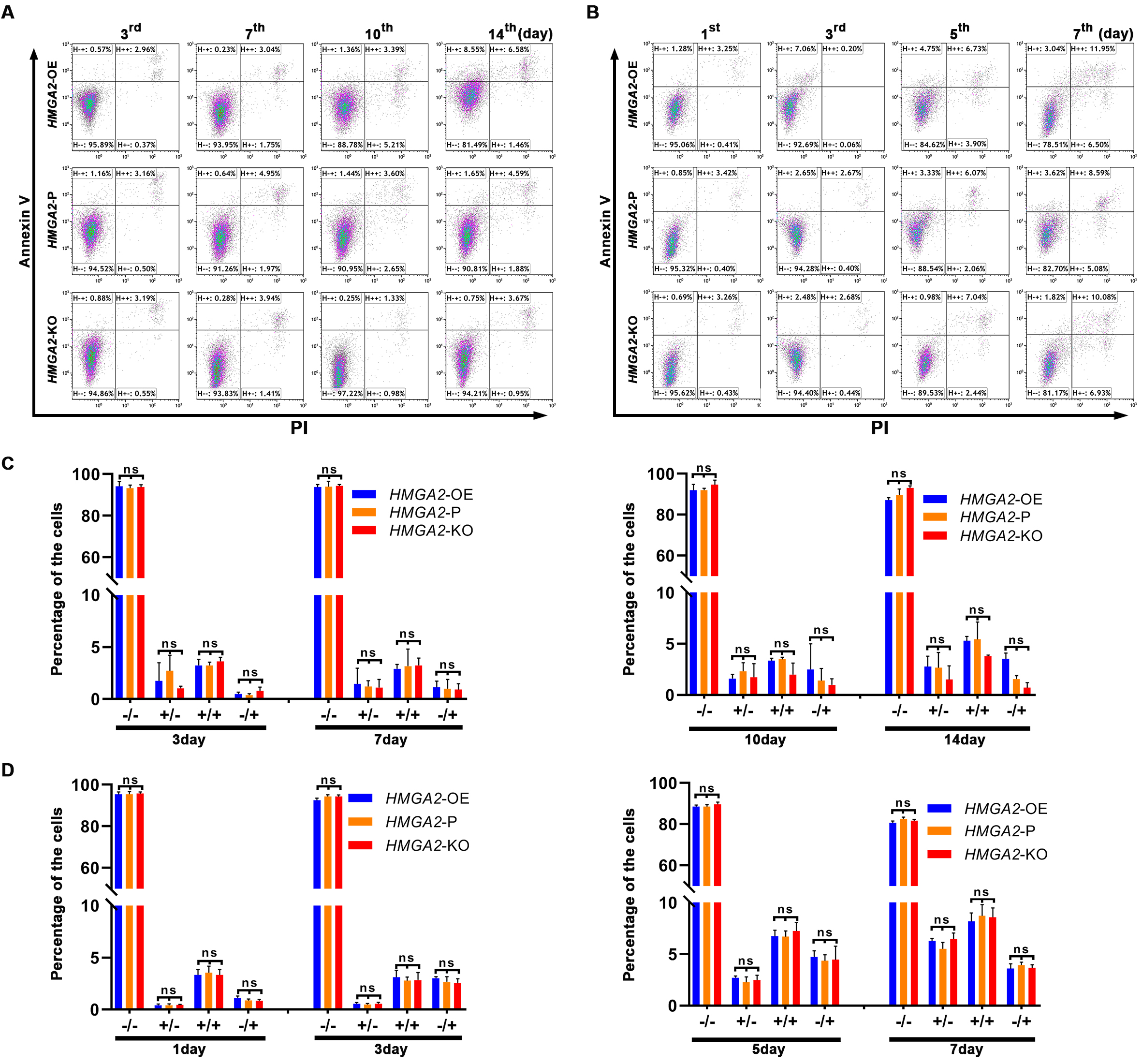

Supplement: Supplementary file 2 [file Image1.JPEG]

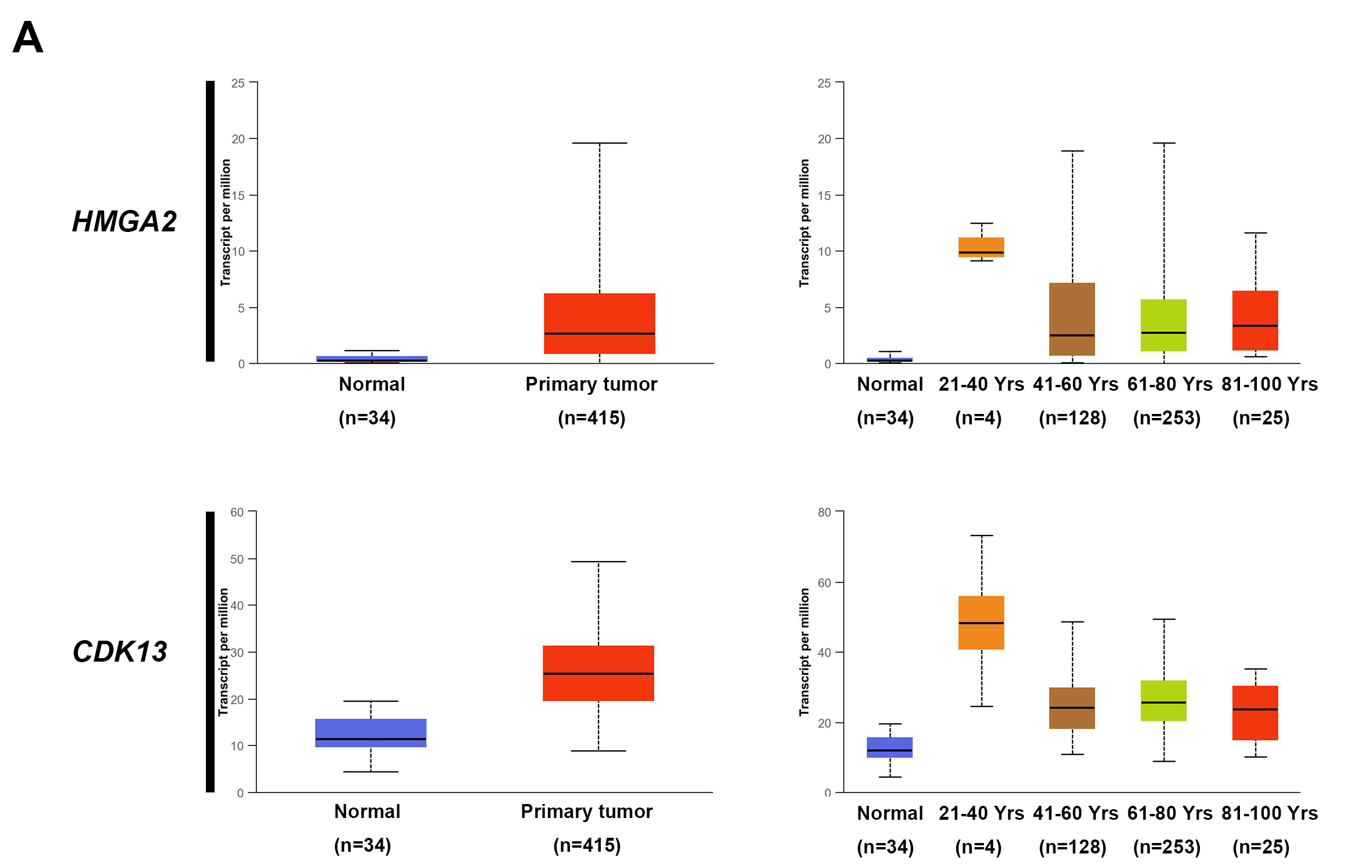

Supplement: Supplementary file 3 [file Image2.JPEG]
